# *N*′-[(*E*)-(4-Bromo-2-thien­yl)methyl­idene]benzohydrazide 0.06-hydrate

**DOI:** 10.1107/S1600536809037350

**Published:** 2009-09-19

**Authors:** Zahid Shafiq, Muhammad Yaqub, M. Nawaz Tahir, Abid Hussain, M. Saeed Iqbal

**Affiliations:** a Department of Chemistry, Bahauddin Zakariya University, Multan 60800, Pakistan; b Department of Physics, University of Sargodha, Sargodha, Pakistan; c Department of Chemistry, Government College University, Lahore, Pakistan

## Abstract

The title compound, C_12_H_9_BrN_2_OS·0.06H_2_O, is a hydrated Schiff base derived from benzoic hydrazide and 4-bromo­thio­phene-2-carboxaldehide. The two Schiff base mol­ecules in the asymmetric unit differ crystallographically: in one mol­ecule the dihedral angle between the benzene ring and thio­phene ring is 49.88 (11)°, whereas the other mol­ecule the rings are almost coplanar with an r.m.s. deviation for the non-H atoms of 0.025 Å. In the crystal, mol­ecules form polymeric sheets linked by N—H⋯O and C—H⋯O hydrogen bonds. The water mol­ecule of crystallization is partially occupied and its H atoms could not be located.

## Related literature

For a related structure, see: Aldoshin *et al.* (1991 ▶).
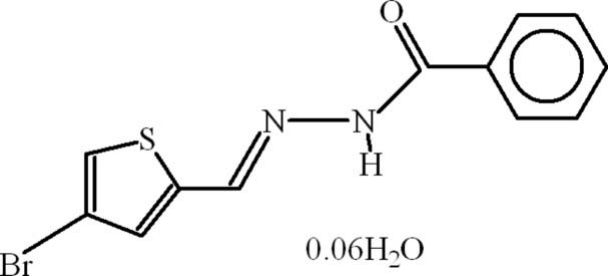


## Experimental

### 

#### Crystal data

C_12_H_9_BrN_2_OS·0.06H_2_O
*M* *_r_* = 310.30
Monoclinic, 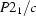

*a* = 8.8348 (4) Å
*b* = 18.3446 (10) Å
*c* = 15.6788 (6) Å
β = 90.274 (2)°
*V* = 2541.1 (2) Å^3^
*Z* = 8
Mo *K*α radiation
μ = 3.38 mm^−1^
*T* = 296 K
0.28 × 0.14 × 0.12 mm

#### Data collection

Bruker Kappa APEXII CCD diffractometer
Absorption correction: multi-scan (*SADABS*; Bruker, 2005 ▶) *T*_min_ = 0.573, *T*_max_ = 0.664
13354 measured reflections
4719 independent reflections
2848 reflections with *I* > 2σ(*I*)
*R*_int_ = 0.048

#### Refinement

*R*[*F* ^2^ > 2σ(*F* ^2^)] = 0.037
*wR*(*F* ^2^) = 0.077
*S* = 1.00
4719 reflections
316 parameters
H-atom parameters constrained
Δρ_max_ = 0.28 e Å^−3^
Δρ_min_ = −0.42 e Å^−3^

### 

Data collection: *APEX2* (Bruker, 2007 ▶); cell refinement: *SAINT* (Bruker, 2007 ▶); data reduction: *SAINT*; program(s) used to solve structure: *SHELXS97* (Sheldrick, 2008 ▶); program(s) used to refine structure: *SHELXL97* (Sheldrick, 2008 ▶); molecular graphics: *ORTEP-3 for Windows* (Farrugia, 1997 ▶) and *PLATON* (Spek, 2009 ▶); software used to prepare material for publication: *WinGX* (Farrugia, 1999 ▶) and *PLATON*.

## Supplementary Material

Crystal structure: contains datablocks global, I. DOI: 10.1107/S1600536809037350/hb5098sup1.cif

Structure factors: contains datablocks I. DOI: 10.1107/S1600536809037350/hb5098Isup2.hkl

Additional supplementary materials:  crystallographic information; 3D view; checkCIF report

## Figures and Tables

**Table 1:** Hydrogen-bond geometry (Å, °)

*D*—H⋯*A*	*D*—H	H⋯*A*	*D*⋯*A*	*D*—H⋯*A*
N1—H1*N*⋯O2	0.86	2.06	2.878 (3)	160
N3—H3*A*⋯O1^i^	0.86	2.12	2.951 (3)	162
C6—H6⋯O2	0.93	2.50	3.231 (4)	136
C20—H20⋯O1^i^	0.93	2.49	3.282 (4)	143

Symmetry code: (i) 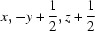.

**Fig. 1.**
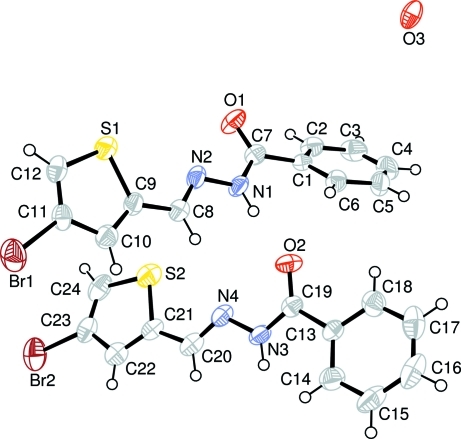
View of (I) with displacement ellipsoids drawn at the 50% probability level. H-atoms are shown by small circles of arbitrary radius.

**Fig. 2.**
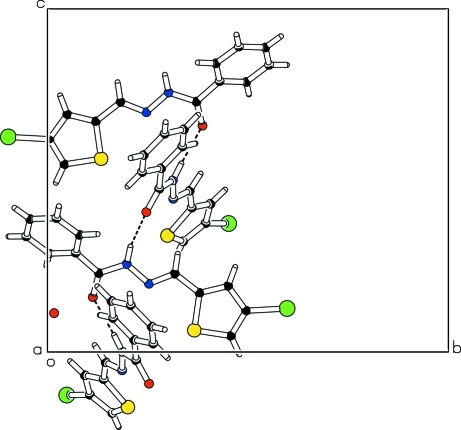
The partial packing of (I) which shows that molecules form polymeric chains which are extending along the crystallographic c axis.

## supplementary crystallographic information

### Comment

The title compound (I, Fig. 1), has been prepared for complexation with various metals and with hope that it will be biologically active as well. The biological studies of (I) are under progress.

The crystal structure of (I) differs from (II) (*N*'-2-(thienylidene))benzhydrazide (Aldoshin *et al.*, 1991) due to bromo substitution.

The title compound consist of two crystallographically different molecules and fractional part of O-atom which occupies the solvent accessible area. The fractional O-atom may be part of methanol, ethanol or water from air. The molecules are stabilized in the form of polymeric sheets due to H-bondings (Table 1, Fig. 2) which extend along the *c* axis. In one molecule the benzene ring A (C1—C6) and the thiophene ring B (C9—C10, S1) are oriented at a dihedral angle of 49.88 (11)° whereas in the other molecule both rings are nearly planar with an r.m.s. deviation of 0.025 Å.

### Experimental

To a hot stirred solution of benzoic hydrazide (1.36 g, 0.01 mol) in ethanol (15 ml) was added 4-bromo-2-thiophencarboxaldehide (1.91 g, 0.01 mol). The resultant mixture was then heated under reflux. After an hour precipitates were formed. The reaction mixture was further heated about 30 min for the completion of the reaction which was monitored through TLC. The reaction mixture was cooled to room temperature, filtered and washed with hot ethanol. The crude material was recrystallized in hot methanol to affoard colourless needles of (I).

### Refinement

There exist solvent accessible volume if only two Schiff base molecules are refined. Therefore, the largest difference peak was taken as a water O-atom and refined anisotropically with an occupancy factor of 0.125. Its presumed H atoms could not be located.

The other H-atoms were positioned geometrically with N—H = 0.86, C—H = 0.93 Å for aromatic H atoms and constrained to ride on their parent atoms, with *U*_iso_(H) = 1.2*U*_eq_(C, N).

### Crystal data

**Table:**

C_12_H_9_BrN_2_OS·0.06H_2_O	*F*(000) = 1236
*M**_r_* = 310.30	*D*_x_ = 1.622 Mg m^−^^3^
Monoclinic, *P*2_1_/*c*	Mo *K*α radiation, λ = 0.71073 Å
Hall symbol: -P 2ybc	Cell parameters from 4719 reflections
*a* = 8.8348 (4) Å	θ = 2.3–25.5°
*b* = 18.3446 (10) Å	µ = 3.38 mm^−^^1^
*c* = 15.6788 (6) Å	*T* = 296 K
β = 90.274 (2)°	Cut needle, colourless
*V* = 2541.1 (2) Å^3^	0.28 × 0.14 × 0.12 mm
*Z* = 8	

### Data collection

**Table:**

Bruker Kappa APEXII CCD diffractometer	4719 independent reflections
Radiation source: fine-focus sealed tube	2848 reflections with *I* > 2σ(*I*)
graphite	*R*_int_ = 0.048
Detector resolution: 7.80 pixels mm^-1^	θ_max_ = 25.5°, θ_min_ = 2.3°
ω scans	*h* = −10→9
Absorption correction: multi-scan (*SADABS*; Bruker, 2005)	*k* = −22→21
*T*_min_ = 0.573, *T*_max_ = 0.664	*l* = −18→18
13354 measured reflections	

### Refinement

**Table:**

Refinement on *F*^2^	Primary atom site location: structure-invariant direct methods
Least-squares matrix: full	Secondary atom site location: difference Fourier map
*R*[*F*^2^ > 2σ(*F*^2^)] = 0.037	Hydrogen site location: inferred from neighbouring sites
*wR*(*F*^2^) = 0.077	H-atom parameters constrained
*S* = 1.00	*w* = 1/[σ^2^(*F*_o_^2^) + (0.0274*P*)^2^ + 0.0751*P*] where *P* = (*F*_o_^2^ + 2*F*_c_^2^)/3
4719 reflections	(Δ/σ)_max_ = 0.001
316 parameters	Δρ_max_ = 0.28 e Å^−^^3^
0 restraints	Δρ_min_ = −0.41 e Å^−^^3^

### Special details

**Table:**

Geometry. Bond distances, angles *etc*. have been calculated using the rounded fractional coordinates. All su's are estimated from the variances of the (full) variance-covariance matrix. The cell e.s.d.'s are taken into account in the estimation of distances, angles and torsion angles
Refinement. Refinement of *F*^2^ against ALL reflections. The weighted *R*-factor *wR* and goodness of fit *S* are based on *F*^2^, conventional *R*-factors *R* are based on *F*, with *F* set to zero for negative *F*^2^. The threshold expression of *F*^2^ > σ(*F*^2^) is used only for calculating *R*-factors(gt) *etc*. and is not relevant to the choice of reflections for refinement. *R*-factors based on *F*^2^ are statistically about twice as large as those based on *F*, and *R*- factors based on ALL data will be even larger.

### Fractional atomic coordinates and isotropic or equivalent isotropic displacement parameters (Å^2^)

**Table:**

	*x*	*y*	*z*	*U*_iso_*/*U*_eq_	Occ. (<1)
Br1	0.14491 (5)	0.59765 (2)	0.12678 (2)	0.0630 (2)	
S1	0.17094 (11)	0.36619 (5)	0.06330 (5)	0.0498 (3)	
O1	0.1624 (3)	0.11321 (12)	0.15983 (13)	0.0533 (9)	
N1	0.2332 (3)	0.19770 (14)	0.25615 (14)	0.0408 (10)	
N2	0.2058 (3)	0.25339 (15)	0.19887 (15)	0.0417 (10)	
C1	0.2702 (3)	0.07061 (17)	0.28937 (17)	0.0336 (11)	
C2	0.1990 (4)	0.00419 (18)	0.28690 (18)	0.0451 (14)	
C3	0.2485 (4)	−0.05279 (19)	0.3370 (2)	0.0556 (14)	
C4	0.3746 (5)	−0.0433 (2)	0.3873 (2)	0.0596 (14)	
C5	0.4483 (4)	0.0227 (2)	0.39020 (19)	0.0510 (16)	
C6	0.3950 (4)	0.08002 (18)	0.34119 (18)	0.0414 (11)	
C7	0.2158 (3)	0.12853 (18)	0.22989 (19)	0.0370 (12)	
C8	0.2124 (3)	0.31748 (18)	0.22827 (19)	0.0424 (11)	
C9	0.1899 (3)	0.37935 (18)	0.17201 (18)	0.0393 (11)	
C10	0.1825 (3)	0.45039 (18)	0.1922 (2)	0.0449 (11)	
C11	0.1601 (3)	0.49531 (17)	0.1202 (2)	0.0396 (11)	
C12	0.1528 (4)	0.45763 (18)	0.0468 (2)	0.0460 (12)	
Br2	−0.53100 (5)	0.45182 (2)	0.37441 (3)	0.0758 (2)	
S2	−0.15815 (11)	0.29933 (6)	0.33933 (5)	0.0559 (4)	
O2	0.3874 (3)	0.24667 (12)	0.40780 (13)	0.0479 (9)	
N3	0.2504 (3)	0.31281 (14)	0.50045 (14)	0.0415 (10)	
N4	0.1274 (3)	0.31373 (15)	0.44655 (14)	0.0434 (11)	
C13	0.5098 (4)	0.28364 (18)	0.53558 (17)	0.0366 (11)	
C14	0.5227 (4)	0.3363 (2)	0.59883 (19)	0.0513 (14)	
C15	0.6512 (4)	0.3403 (2)	0.6488 (2)	0.0640 (18)	
C16	0.7682 (4)	0.2919 (3)	0.6359 (2)	0.0684 (19)	
C17	0.7573 (4)	0.2400 (2)	0.5739 (3)	0.0623 (17)	
C18	0.6284 (4)	0.23582 (18)	0.5242 (2)	0.0497 (14)	
C19	0.3774 (4)	0.27852 (17)	0.47561 (19)	0.0360 (12)	
C20	0.0191 (4)	0.3547 (2)	0.47003 (19)	0.0494 (16)	
C21	−0.1191 (4)	0.35979 (19)	0.42087 (18)	0.0451 (11)	
C22	−0.2347 (4)	0.4067 (2)	0.4320 (2)	0.0497 (14)	
C23	−0.3549 (4)	0.3943 (2)	0.3754 (2)	0.0490 (14)	
C24	−0.3311 (4)	0.3383 (2)	0.3225 (2)	0.0564 (16)	
O3	0.911 (2)	0.0164 (9)	0.1129 (9)	0.048 (7)	0.125
H1N	0.26089	0.20713	0.30764	0.0489*	
H2	0.11612	−0.00246	0.25091	0.0537*	
H3	0.19721	−0.09707	0.33675	0.0669*	
H4	0.41062	−0.08202	0.41992	0.0714*	
H5	0.53323	0.02878	0.42476	0.0612*	
H6	0.44347	0.12498	0.34324	0.0496*	
H8	0.23166	0.32497	0.28597	0.0509*	
H10	0.19111	0.46807	0.24758	0.0536*	
H12	0.13932	0.47887	−0.00657	0.0548*	
H3A	0.24631	0.33386	0.54939	0.0496*	
H14	0.44400	0.36913	0.60754	0.0619*	
H15	0.65900	0.37564	0.69119	0.0765*	
H16	0.85492	0.29467	0.66954	0.0818*	
H17	0.83660	0.20759	0.56515	0.0746*	
H18	0.62129	0.20009	0.48232	0.0594*	
H20	0.02921	0.38194	0.51978	0.0592*	
H22	−0.23469	0.44331	0.47297	0.0597*	
H24	−0.40030	0.32254	0.28156	0.0676*	

### Atomic displacement parameters (Å^2^)

**Table:**

	*U*^11^	*U*^22^	*U*^33^	*U*^12^	*U*^13^	*U*^23^
Br1	0.0723 (3)	0.0383 (2)	0.0784 (3)	−0.0020 (2)	−0.0096 (2)	0.0086 (2)
S1	0.0682 (7)	0.0444 (6)	0.0368 (5)	0.0061 (5)	−0.0025 (4)	0.0047 (4)
O1	0.0733 (18)	0.0508 (16)	0.0357 (13)	−0.0143 (13)	−0.0168 (12)	0.0050 (11)
N1	0.0569 (19)	0.0380 (18)	0.0274 (14)	0.0006 (14)	−0.0068 (12)	0.0069 (13)
N2	0.0497 (19)	0.0413 (18)	0.0340 (15)	0.0028 (14)	−0.0042 (12)	0.0099 (14)
C1	0.037 (2)	0.038 (2)	0.0259 (17)	0.0035 (16)	0.0034 (14)	−0.0021 (14)
C2	0.055 (3)	0.042 (2)	0.0382 (19)	−0.0052 (18)	0.0015 (16)	−0.0039 (17)
C3	0.085 (3)	0.031 (2)	0.051 (2)	−0.007 (2)	0.013 (2)	−0.0002 (18)
C4	0.087 (3)	0.040 (2)	0.052 (2)	0.021 (2)	0.010 (2)	0.0125 (18)
C5	0.052 (3)	0.056 (3)	0.045 (2)	0.013 (2)	−0.0027 (17)	0.0107 (18)
C6	0.042 (2)	0.042 (2)	0.0403 (19)	−0.0007 (16)	0.0022 (16)	0.0043 (16)
C7	0.040 (2)	0.038 (2)	0.033 (2)	−0.0064 (16)	0.0003 (16)	0.0030 (16)
C8	0.050 (2)	0.042 (2)	0.0351 (19)	0.0037 (17)	−0.0027 (15)	0.0030 (17)
C9	0.043 (2)	0.040 (2)	0.0349 (19)	0.0040 (16)	−0.0029 (15)	0.0067 (15)
C10	0.048 (2)	0.044 (2)	0.0427 (19)	0.0001 (18)	−0.0074 (16)	−0.0004 (18)
C11	0.038 (2)	0.0307 (19)	0.050 (2)	−0.0035 (15)	−0.0044 (16)	0.0094 (17)
C12	0.048 (2)	0.045 (2)	0.045 (2)	0.0010 (18)	−0.0016 (16)	0.0156 (17)
Br2	0.0563 (3)	0.0776 (3)	0.0934 (3)	0.0089 (2)	−0.0164 (2)	0.0157 (2)
S2	0.0563 (7)	0.0660 (7)	0.0454 (5)	−0.0039 (5)	−0.0074 (4)	−0.0029 (5)
O2	0.0560 (16)	0.0536 (16)	0.0340 (12)	−0.0029 (12)	0.0005 (11)	−0.0139 (12)
N3	0.0431 (19)	0.054 (2)	0.0273 (15)	0.0011 (14)	−0.0061 (13)	−0.0039 (12)
N4	0.0431 (19)	0.057 (2)	0.0301 (16)	−0.0010 (15)	−0.0055 (14)	0.0019 (13)
C13	0.041 (2)	0.041 (2)	0.0277 (18)	−0.0029 (16)	−0.0016 (15)	0.0054 (15)
C14	0.046 (2)	0.066 (3)	0.042 (2)	0.0023 (19)	−0.0008 (17)	−0.0057 (18)
C15	0.055 (3)	0.097 (4)	0.040 (2)	−0.010 (2)	−0.0129 (19)	−0.006 (2)
C16	0.051 (3)	0.100 (4)	0.054 (3)	−0.010 (3)	−0.017 (2)	0.027 (2)
C17	0.053 (3)	0.063 (3)	0.071 (3)	0.007 (2)	−0.002 (2)	0.026 (2)
C18	0.056 (3)	0.042 (2)	0.051 (2)	0.0013 (19)	0.0012 (19)	0.0075 (17)
C19	0.042 (2)	0.034 (2)	0.032 (2)	−0.0060 (16)	0.0004 (16)	0.0047 (15)
C20	0.053 (3)	0.061 (3)	0.034 (2)	−0.005 (2)	−0.0064 (18)	0.0022 (17)
C21	0.050 (2)	0.054 (2)	0.0313 (19)	−0.0032 (19)	−0.0050 (17)	0.0022 (16)
C22	0.051 (3)	0.056 (2)	0.042 (2)	−0.002 (2)	−0.0031 (18)	0.0011 (18)
C23	0.048 (2)	0.056 (3)	0.043 (2)	−0.0053 (19)	−0.0058 (17)	0.0147 (19)
C24	0.050 (3)	0.070 (3)	0.049 (2)	−0.016 (2)	−0.0162 (17)	0.011 (2)
O3	0.058 (13)	0.047 (12)	0.039 (10)	−0.005 (9)	−0.010 (8)	0.021 (8)

### Geometric parameters (Å, °)

**Table:**

Br1—C11	1.885 (3)	C2—H2	0.9300
Br2—C23	1.880 (4)	C3—H3	0.9300
S1—C9	1.729 (3)	C4—H4	0.9300
S1—C12	1.705 (3)	C5—H5	0.9300
S2—C21	1.726 (3)	C6—H6	0.9300
S2—C24	1.706 (4)	C8—H8	0.9300
O1—C7	1.226 (4)	C10—H10	0.9300
O2—C19	1.217 (4)	C12—H12	0.9300
N1—N2	1.381 (4)	C13—C14	1.389 (4)
N1—C7	1.343 (4)	C13—C19	1.500 (5)
N2—C8	1.264 (4)	C13—C18	1.379 (5)
N1—H1N	0.8600	C14—C15	1.378 (5)
N3—N4	1.374 (4)	C15—C16	1.378 (6)
N3—C19	1.346 (4)	C16—C17	1.364 (6)
N4—C20	1.273 (4)	C17—C18	1.379 (5)
N3—H3A	0.8600	C20—C21	1.444 (5)
C1—C2	1.372 (5)	C21—C22	1.348 (5)
C1—C7	1.492 (4)	C22—C23	1.399 (5)
C1—C6	1.377 (4)	C23—C24	1.338 (5)
C2—C3	1.378 (5)	C14—H14	0.9300
C3—C4	1.373 (5)	C15—H15	0.9300
C4—C5	1.375 (5)	C16—H16	0.9300
C5—C6	1.384 (5)	C17—H17	0.9300
C8—C9	1.451 (4)	C18—H18	0.9300
C9—C10	1.343 (5)	C20—H20	0.9300
C10—C11	1.411 (4)	C22—H22	0.9300
C11—C12	1.344 (4)	C24—H24	0.9300
			
C9—S1—C12	91.19 (16)	N2—C8—H8	120.00
C21—S2—C24	91.20 (17)	C9—C10—H10	124.00
N2—N1—C7	118.7 (2)	C11—C10—H10	124.00
N1—N2—C8	116.3 (2)	S1—C12—H12	124.00
N2—N1—H1N	121.00	C11—C12—H12	124.00
C7—N1—H1N	121.00	C14—C13—C19	123.5 (3)
N4—N3—C19	119.1 (2)	C14—C13—C18	118.4 (3)
N3—N4—C20	115.0 (2)	C18—C13—C19	118.0 (3)
C19—N3—H3A	120.00	C13—C14—C15	120.5 (3)
N4—N3—H3A	120.00	C14—C15—C16	119.9 (3)
C6—C1—C7	122.3 (3)	C15—C16—C17	120.3 (3)
C2—C1—C6	119.6 (3)	C16—C17—C18	119.8 (3)
C2—C1—C7	118.0 (3)	C13—C18—C17	121.2 (3)
C1—C2—C3	120.9 (3)	O2—C19—C13	121.2 (3)
C2—C3—C4	119.1 (3)	O2—C19—N3	122.8 (3)
C3—C4—C5	120.9 (3)	N3—C19—C13	116.0 (3)
C4—C5—C6	119.4 (3)	N4—C20—C21	121.3 (3)
C1—C6—C5	120.1 (3)	S2—C21—C20	121.3 (3)
N1—C7—C1	116.5 (3)	C20—C21—C22	127.7 (3)
O1—C7—C1	121.2 (3)	S2—C21—C22	110.9 (3)
O1—C7—N1	122.3 (3)	C21—C22—C23	112.8 (3)
N2—C8—C9	120.0 (3)	Br2—C23—C22	122.7 (3)
S1—C9—C8	120.2 (2)	Br2—C23—C24	124.0 (3)
C8—C9—C10	128.5 (3)	C22—C23—C24	113.4 (3)
S1—C9—C10	111.3 (2)	S2—C24—C23	111.7 (3)
C9—C10—C11	112.7 (3)	C13—C14—H14	120.00
C10—C11—C12	113.0 (3)	C15—C14—H14	120.00
Br1—C11—C10	123.2 (2)	C14—C15—H15	120.00
Br1—C11—C12	123.8 (2)	C16—C15—H15	120.00
S1—C12—C11	111.9 (2)	C15—C16—H16	120.00
C3—C2—H2	120.00	C17—C16—H16	120.00
C1—C2—H2	120.00	C16—C17—H17	120.00
C4—C3—H3	120.00	C18—C17—H17	120.00
C2—C3—H3	120.00	C13—C18—H18	119.00
C5—C4—H4	120.00	C17—C18—H18	119.00
C3—C4—H4	120.00	N4—C20—H20	119.00
C4—C5—H5	120.00	C21—C20—H20	119.00
C6—C5—H5	120.00	C21—C22—H22	124.00
C1—C6—H6	120.00	C23—C22—H22	124.00
C5—C6—H6	120.00	S2—C24—H24	124.00
C9—C8—H8	120.00	C23—C24—H24	124.00
			
C12—S1—C9—C8	−179.4 (2)	N2—C8—C9—C10	175.0 (3)
C12—S1—C9—C10	−0.1 (2)	S1—C9—C10—C11	0.5 (3)
C9—S1—C12—C11	−0.3 (3)	C8—C9—C10—C11	179.7 (3)
C24—S2—C21—C22	−0.5 (3)	C9—C10—C11—Br1	−179.94 (19)
C21—S2—C24—C23	0.8 (3)	C9—C10—C11—C12	−0.8 (4)
C24—S2—C21—C20	176.9 (3)	Br1—C11—C12—S1	179.84 (17)
C7—N1—N2—C8	174.8 (3)	C10—C11—C12—S1	0.7 (4)
N2—N1—C7—O1	−7.3 (4)	C18—C13—C14—C15	−0.1 (5)
N2—N1—C7—C1	170.5 (2)	C19—C13—C14—C15	−176.5 (3)
N1—N2—C8—C9	178.0 (2)	C14—C13—C18—C17	−0.3 (5)
C19—N3—N4—C20	−171.6 (3)	C19—C13—C18—C17	176.4 (3)
N4—N3—C19—O2	−1.6 (5)	C14—C13—C19—O2	159.0 (3)
N4—N3—C19—C13	176.5 (3)	C14—C13—C19—N3	−19.1 (5)
N3—N4—C20—C21	−178.4 (3)	C18—C13—C19—O2	−17.4 (5)
C6—C1—C2—C3	1.5 (5)	C18—C13—C19—N3	164.4 (3)
C7—C1—C2—C3	176.7 (3)	C13—C14—C15—C16	0.2 (5)
C2—C1—C6—C5	0.2 (5)	C14—C15—C16—C17	−0.1 (6)
C7—C1—C6—C5	−174.8 (3)	C15—C16—C17—C18	−0.3 (6)
C2—C1—C7—O1	−31.2 (4)	C16—C17—C18—C13	0.4 (6)
C6—C1—C7—N1	−34.0 (4)	N4—C20—C21—S2	11.7 (5)
C2—C1—C7—N1	150.9 (3)	N4—C20—C21—C22	−171.4 (3)
C6—C1—C7—O1	143.9 (3)	S2—C21—C22—C23	0.1 (4)
C1—C2—C3—C4	−2.6 (5)	C20—C21—C22—C23	−177.1 (3)
C2—C3—C4—C5	2.1 (5)	C21—C22—C23—Br2	−179.1 (3)
C3—C4—C5—C6	−0.5 (5)	C21—C22—C23—C24	0.5 (4)
C4—C5—C6—C1	−0.7 (5)	Br2—C23—C24—S2	178.73 (19)
N2—C8—C9—S1	−5.8 (4)	C22—C23—C24—S2	−0.9 (4)

### Hydrogen-bond geometry (Å, °)

**Table:**

*D*—H···*A*	*D*—H	H···*A*	*D*···*A*	*D*—H···*A*
N1—H1N···O2	0.86	2.06	2.878 (3)	160
N3—H3A···O1^i^	0.86	2.12	2.951 (3)	162
C6—H6···O2	0.93	2.50	3.231 (4)	136
C20—H20···O1^i^	0.93	2.49	3.282 (4)	143

Symmetry codes: (i) *x*, −*y*+1/2, *z*+1/2.
